# Let’s Get Physical: Flavivirus-Host Protein–Protein Interactions in Replication and Pathogenesis

**DOI:** 10.3389/fmicb.2022.847588

**Published:** 2022-03-03

**Authors:** Adam T. Fishburn, Oanh H. Pham, Matthew W. Kenaston, Nitin S. Beesabathuni, Priya S. Shah

**Affiliations:** ^1^Department of Microbiology and Molecular Genetics, University of California, Davis, Davis, CA, United States; ^2^Department of Chemical Engineering, University of California, Davis, Davis, CA, United States

**Keywords:** flavivirus, protein–protein interactions, virus replication, virus pathogenesis, virus host interactions, autophagy

## Abstract

Flaviviruses comprise a genus of viruses that pose a significant burden on human health worldwide. Transmission by both mosquito and tick vectors, and broad host tropism contribute to the presence of flaviviruses globally. Like all viruses, they require utilization of host molecular machinery to facilitate their replication through physical interactions. Their RNA genomes are translated using host ribosomes, synthesizing viral proteins that cooperate with each other and host proteins to reshape the host cell into a factory for virus replication. Thus, dissecting the physical interactions between viral proteins and their host protein targets is essential in our comprehension of how flaviviruses replicate and how they alter host cell behavior. Beyond replication, even single interactions can contribute to immune evasion and pathogenesis, providing potential avenues for therapeutic intervention. Here, we review protein interactions between flavivirus and host proteins that contribute to virus replication, immune evasion, and disease.

## Introduction

*Flavivirus* is a genus of positive-sense, single-stranded RNA (ssRNA+), arthropod-transmitted viruses within the family *Flaviviridae*. The ssRNA genome contains a single open-reading frame, which is translated by host ribosomes into a large viral polyprotein. This polyprotein is co-translationally processed by viral and host proteases into 10 individual viral proteins. Three of these proteins are referred to as structural proteins which include Capsid (C), pre-Membrane (prM), and Envelope (Env) proteins, which form the physical virion. The remaining seven proteins are referred to as non-structural (NS) proteins, which include NS1, NS2A, NS2B, NS3, NS4A, NS4B, and NS5. These proteins are not components of infectious virions but rather play broad roles within infected cells in generating virus progeny. Four distinct enzymatic activities are encoded within two NS proteins. NS3 serves as the helicase. It also interacts with NS2B as a cofactor (NS2B3) to form the viral protease (Falgout et al., 1991). NS5 is both the RNA-dependent RNA-polymerase and methyltransferase, which synthesizes and caps new RNA genomes (Tan et al., 1996; Egloff et al., 2002; Ray et al., 2006). The RNA genome also contains 3' and 5' untranslated regions (UTRs) with loop-like structures that play roles in genome stability and translation (Alvarez et al., 2005; Lodeiro et al., 2009). Genome replication occurs within the remodeled ER in involuted structures referred to as virus replication organelles or replication compartments (Gillespie et al., 2010). These substructures serve to concentrate replication substrates and hide viral nucleic acids from detection by the host immune response. Here, the viral NS proteins assemble into the replication complex, which performs the enzymatic steps of RNA synthesis (Welsch et al., 2009; Yi et al., 2012). Viral ssRNA+ is initially used as a template for the synthesis of negative-sense ssRNA (ssRNA-), which in turn is used as a template to synthesize more ssRNA+. As replication progresses these genomes are either further amplified or packaged into progeny virions. In addition to genome replication by the replication complex, viral NS proteins mediate different aspects of virus replication, such as ER remodeling and modulating the host immune response.

The most well-studied flaviviruses are those that cause significant disease in humans. For mosquito-transmitted viruses this includes dengue virus (DENV), Zika virus (ZIKV), West Nile virus (WNV), yellow fever virus (YFV), and Japanese encephalitis virus (JEV). These flaviviruses are all transmitted by mosquitoes of either *Aedes* or *Culex* spp. (Huang et al., 2014). DENV is the most widespread and threatening flavivirus. Currently, there are four well-described serotypes of DENV, referred to as DENV1–DENV4, that each have distinct molecular and physiological characteristics (Chandramouli et al., 2010; Yung et al., 2015). World-wide there are an estimated 390 million cases of DENV infection per year, occurring across 128 countries, although most infections occur in Asia (Brady et al., 2012; Bhatt et al., 2013). Recently, the emergence of a fifth DENV serotype (DENV5) with a sylvatic replication cycle has been reported (da Silva Voorham, 2014; Mustafa et al., 2015). However, DENV5 remains a controversial topic, as the evidence to support the existence of this serotype is limited and mathematical modeling suggests a low probability for the emergence of new DENV serotypes (Sánchez-González et al., 2021). ZIKV recently received major research due to the 2015–2016 epidemic and the revelation that congenital ZIKV infection causes birth defects, collectively referred to as congenital Zika syndrome (CZS; de Araújo et al., 2016; Moore et al., 2017). ZIKV infection in adults is usually limited to mild flu-like illness but can be rarely associated with Guillain-Barré Syndrome, a condition where nerves are damaged, usually in the extremities (Cao-Lormeau et al., 2016). While less common, WNV and JEV can also cause encephalitis (Solomon et al., 2000; DeBiasi and Tyler, 2006). Tick-borne flaviviruses are transmitted by many different ticks, including *Haemaphysalis*, *Ixodes*, *Dermacentor*, and *Ornithidoros* spp. (de la Fuente et al., 2017). These account for much fewer total human infections, many of which are in vastly different geographical settings compared to the tropical climates which host mosquitoes. The most notable of these are tick-borne encephalitis virus (TBEV) and Powassan virus (POWV). While the number of human infections arising from tick-borne viruses is relatively limited the resulting disease can be very severe. Encephalitis resulting from TBEV infection can appear in several forms, with an overall mortality rate of around 2% (Ruzek et al., 2019). Given the severity of disease caused by flaviviruses, it is critical to understand mechanisms of replication and pathogenesis.

**Figure 1:**
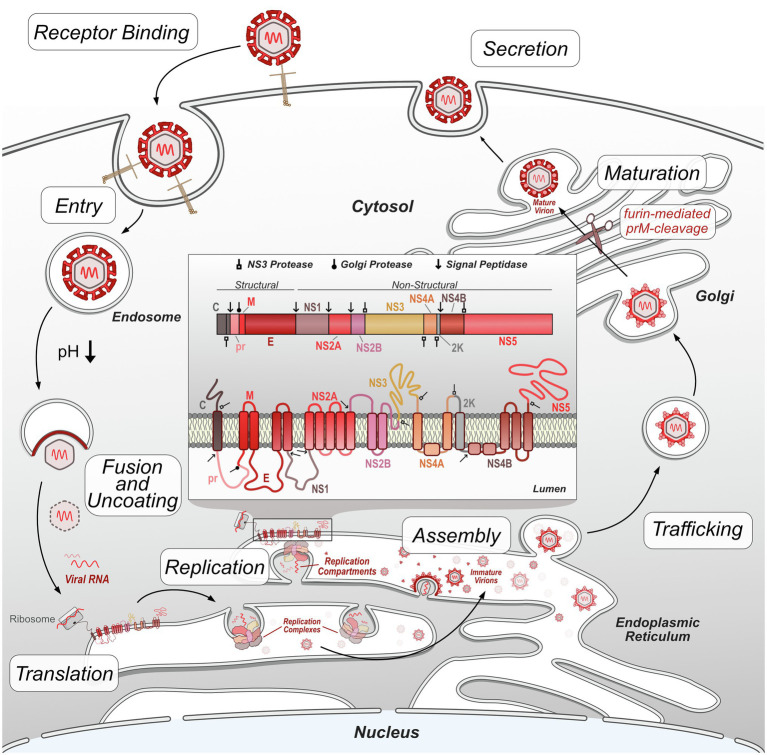
Flavivirus replication cycle. Flavivirus infection begins by receptor-mediated binding to the host cell and entry *via* clathrin-mediated endocytosis. Decreases in endosome pH trigger virion envelope fusion with the endosome membrane, releasing the genome into the host cytosol. After uncoating, the viral RNA genome is translated by host ribosomes into the viral polypeptide, which is co-translationally processed, including insertion of transmembrane proteins into the ER and cleavage of the polypeptide by host and viral proteases into individual proteins. Non-structural viral proteins form replication complexes, which replicate viral RNA genomes within invaginated ER compartments. Structural viral proteins are assembled and loaded with viral genetic material in the ER prior to entering the trans-Golgi network. In the Golgi, immature virions are processed by furin protease cleavage of prM, resulting in mature, infectious virions. These virions exit the cell by exocytosis and continue the replication cycle by initiating infection of other host cells.

In general, flaviviruses have a conserved replication cycle, which includes viral entry, virion fusion with the endosome and release of viral RNA, genome replication and protein production in the ER, virion packaging and processing through the secretory pathway, and viral release *via* exocytosis (Figure 1). At each of these stages, flaviviruses are dependent on host machinery to perform necessary functions. The limited flavivirus genome size requires them to maximize the functions of each protein they encode. Flavivirus replication is therefore largely dependent on the interactions between viral proteins and host proteins to manipulate their biology through direct and indirect mechanisms. These protein interactions can be identified using targeted and comprehensive screening approaches (Coyaud et al., 2018; Scaturro et al., 2018; Shah et al., 2018; Cao et al., 2019; Li et al., 2019; Wang et al., 2019; Golubeva et al., 2020; Tan et al., 2020; Zeng et al., 2020; Lemasson et al., 2021). This review will focus on virus-host protein–protein interactions (PPIs) emerging from both targeted and comprehensive studies that directly facilitate flavivirus replication, dampen host immune response, or disrupt cellular processes to cause disease. While not covered here, it is worth noting that additional virus-host interactions, such as RNA-protein, and RNA–RNA interactions also play important roles in flavivirus replication and disease (Funk et al., 2010; Chavali et al., 2017; Damas et al., 2019; Ooi et al., 2019).

## Flavivirus-Host PPIs Facilitate Fundamental Aspects of Flavivirus Replication

In this section, we review the data emerging from both comprehensive and targeted studies of flavivirus-host PPIs as they relate to various stages of flavivirus replication.

### Virus Attachment Factors

**Figure 2:**
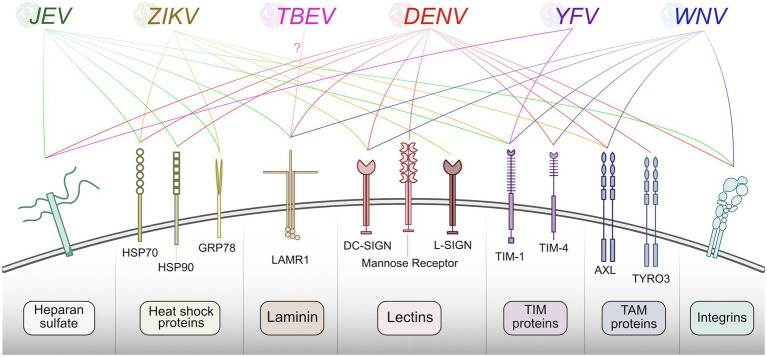
Summary of host proteins used by flaviviruses for entry. Flaviviruses recognize and bind plasma membrane host factors to initiate entry into the host cell. Different flaviviruses utilize a similar pool of host proteins for entry.

The first step in any virus replication cycle is entry into the host cell and involves the classic virus-host protein interaction between a virion structural protein and a host attachment factor. In the case of flaviviruses, Env proteins on the virion exterior interact and attach to host factors on the plasma membrane surface. Flavivirus Env proteins are quite promiscuous and can bind many different host factors. While each flavivirus appears to bind multiple host factors, not all flaviviruses use the same set of host factors for entry. Generally, flaviviruses use TAM (e.g., Tyro3, Axl, and Mer) family receptor tyrosine kinases (Meertens et al., 2012; Richard et al., 2017), phosphatidyl serine receptor T-cell immunoglobulin (TIM; Dejarnac et al., 2018; Niu et al., 2018; Zhang et al., 2022), C-type lectin receptors (e.g., DC-SIGN; Miller et al., 2008; Pereira et al., 2019; Routhu et al., 2019), integrins (Chu and Ng, 2004; Schmidt et al., 2013), heat-shock proteins 70/90 (Reyes-Del Valle et al., 2005; Das et al., 2009; Pujhari et al., 2019), laminin receptor (LAMR1; Tio et al., 2005; Thongtan et al., 2012), and heparan sulfate (Chen et al., 1997; Germi et al., 2002) as means of attachment. Subtle differences in Env protein sequence likely contribute to differences in host factor usage, and distinct tissue tropisms between flaviviruses. The well-established flavivirus-host attachment factors are described in Figure 2; however, it is essential to note that there are likely others that each virus uses that have not been identified.

Interestingly, some entry determinants are dependent on specific intracellular virus-host interactions that provide newly generated progeny virions with additional receptor targets. A recent study elegantly showed how the interaction between TRIM7, an E3-ubiquitin ligase, and Env resulted in specific polyubiquitination in the infected cell that allowed progeny virion binding to Tim-1 of the new target cell (Giraldo et al., 2020). Here, ZIKV Env was ubiquitinated on three lysine residues: K38, K63, and K281. A recombinant virus in which one of these lysines was swapped with arginine (E-K38R) was significantly attenuated in JEG-3 placental trophoblast cells and *in vivo* in mice, but not in mosquitoes. Intriguingly, the ZIKV E-K38R titers *in vivo* varied significantly by tissue, suggesting that Env ubiquitination may drive tissue specific tropism. To further explore this, they generated TRIM7 knockout cells which attenuated ZIKV replication in JEG-3 cells but did not affect DENV replication in A549 lung epithelial cells. Similar results were observed in *Trim7^−/−^* knockout mice. In this model, ZIKV replicated similarly to WT in the heart, liver, lung, and muscle, whereas ZIKV replication in the brain, eyes, and reproductive tissues was significantly reduced. Finally, they identified that Tim-1 interacted with wild-type ZIKV but very minimally with K38R viral particles, suggesting that ubiquitination at this site is critical for the interaction and virus entry through Tim-1. This was supported both by reduced attachment of ZIKV to Tim-1 knockout JEG-3 cells and reduced replication of ZIKV in the brains of *Tim1^−/−^* knockout mice (Giraldo et al., 2020). All together, these results indicate an important interaction between ZIKV Env and TRIM7, providing ubiquitination that mediates entry into the brain and other tissues that are major contributors to ZIKV pathogenesis. Currently, it is unknown if other flaviviruses use TRIM7 ubiquitination of Env to mediate entry *via* TIM-1, as many other flaviviruses use TIM-1 as a host receptor (Meertens et al., 2012; Dejarnac et al., 2018; Niu et al., 2018; Zhang et al., 2022).

Intriguingly, a recent study identified this same ZIKV Env ubiquitination is also targeted by host factors and restricts virus infection (Hu et al., 2021). The laminin receptor LAMR1 consists of an intracellular domain, a transmembrane domain, and a larger extracellular domain, which is known to be utilized as an attachment factor by several flaviviruses that are not ZIKV (Tio et al., 2005; Sakoonwatanyoo et al., 2006; Bogachek et al., 2008; Thongtan et al., 2012). Unsurprisingly, ZIKV Env was also found to interact with LAMR1. However, it only interacts with the intracellular region, not the extracellular region that would mediate extracellular virion attachment. Overexpression of LAMR1 reduced virus replication and repression of LAMR1 by shRNA resulted in significant increases in viral titer. The interaction between ZIKV Env and LAMR1 is mediated by a single amino acid in Env, G282. Interestingly, G282 is very highly conserved among ZIKV strains, but is not conserved at all with other flaviviruses. Further, the authors found that LAMR1 recruits eukaryotic translation initiation factor 3 subunit 5 (EIF3S5), a member of the ubiquitin proteasome system (UPS). Knockdown of EIF3S5 reduced Env deubiquitination and increased the levels of NS5, Env, and viral RNA in infected HeLa cells (Hu et al., 2021). Thus, Env ubiquitination can have opposing effects mediated by different Env-host protein interactions.

It is worth noting that there is plentiful information on the host entry factors of mosquito-borne flaviviruses. However, knowledge on the attachment factors utilized by tick-borne flaviviruses is extremely limited. One recent study attempted to identify attachment factors for Langat virus (LGTV). They found that LGTV did not utilize heparin sulfate, *O-* or *N-*linked glycans, or glycolipids for entry, suggesting that the host receptor is protein in nature. However, they were unable to definitively identify such a protein (Rodrigues et al., 2019). A pair of studies suggests one such attachment factor might be LAMR1, although additional studies are required (Protopopova et al., 1997; Malygin et al., 2009). Another recently published study used multiple methods to identify TIM-1 as an entry factor for TBEV (Zhang et al., 2022).

### Fusion and Uncoating

After a flavivirus binds to an extracellular host entry factor, it enters the intracellular space by clathrin-mediated endocytosis (Acosta et al., 2008; van der Schaar et al., 2008). There is several cargo internalization factors involved in this process that are necessary for flavivirus infection. Specifically, LY6E has been shown to reorganize itself into tubule-like structures to support entry of WNV, ZIKV, and DENV (Hackett and Cherry, 2018), though direct virus-host interactions have yet to be identified in this case. Following endocytosis, the membrane of the virus envelope must fuse with the endosome membrane. This process happens through a mechanism not requiring any physical virus-host protein interactions. V-ATPase pumps protons from the cytoplasm into the lumen of various organelles, including endosomes, decreasing the intra-endosomal pH (Lafourcade et al., 2008; Kozik et al., 2013). This triggers conformational changes in viral Env proteins that ultimately lead to their insertion into the endosome membrane and formation of the fusion pore, releasing the nucleocapsid to the cytosol (Allison et al., 1995; Chao et al., 2014). Once released the viral genome is not immediately capable of being translated. It first must be stripped of the capsid proteins that otherwise stabilize and protect viral RNA. This occurs through the ubiquitination of Capsid proteins by host UBA1 (Byk et al., 2016), with subsequent nucleocapsid disassembly shown to be mediated by VCP (Ramanathan et al., 2020). Once uncoated, the flavivirus RNA genome may be translated into the viral polyprotein.

## Interactions Involved in Viral Protein Translation and Stability

**Figure 3:**
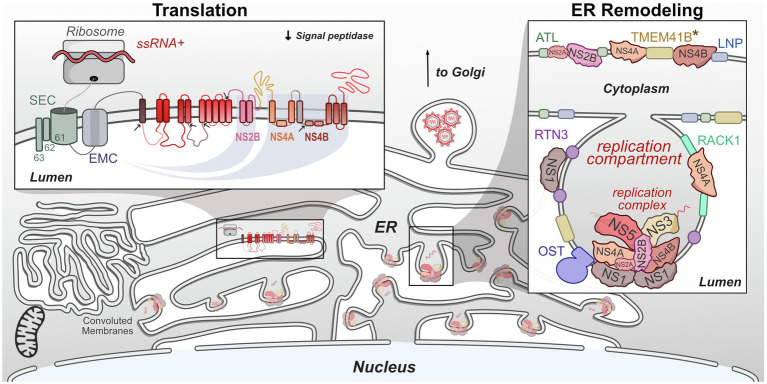
Flaviviruses co-opt host proteins to remodel the ER. Flaviviruses dramatically alter the morphology of the host ER to create a niche that maximizes the efficiency of genome replication and virion packaging. Replication compartments are formed by the involution of the ER membrane by both viral and host proteins. Viral replication complexes reside within these compartments and carry out RNA replication. These complexes also physically associate with host proteins. Viral single-stranded RNA (ssRNA)+ genomes are translated on the ER by host ribosomes. The resulting viral polyprotein is co-translationally processed to ensure its stability, insertion into the ER membrane, and proper cleavage into individual viral proteins. *TMEM41B is known to interact with either ZIKV NS4A or YFV NS4B and may facilitate ER remodeling.

Translation of flavivirus genomes into functional viral proteins is dependent on the activity of several host pathways. As is true of all viruses, host ribosomes are required to initially translate the genome into the viral polyprotein. Several host proteases are necessary for polyprotein cleavage into singular proteins. However, many of these NS proteins contain multiple transmembrane domains, specifically NS2A, NS2B, NS4A, and NS4B, which all must be correctly inserted into the ER membrane in the correct orientation in order to be functional (Miller et al., 2007; Xie et al., 2013; Li et al., 2015, 2016). The stability and insertion of these proteins are performed by the signal-recognition particle (SRP), host SEC61 translocon, and ER membrane complex (EMC). These complexes have been shown to be critical host factors for many viruses, including flaviviruses (Sessions et al., 2009; Heaton et al., 2016; Marceau et al., 2016; Zhang et al., 2016). In this section, we will review recent work that has advanced our understanding of how flaviviruses co-opt these complexes during infection (Figure 3).

### SRP-Translocon Pathway

Flaviviruses replicate within the ER and utilize ER-associated ribosomes for translation. The translated polyprotein contains many transmembrane domains that must be properly integrated into the ER membrane. This function is performed by the SRP-translocon pathway, in which the SRP ribonucleotide complex binds and identifies a hydrophobic transmembrane region of the nascent polypeptide, arrests translation, and brings the ribosome to a translocon where translation continues (Keenan et al., 2001). In eukaryotes, the Sec translocon is made up of the SEC61 complex (SEC61α/β/γ), SEC62/63, and a number of other proteins that can vary depending on substrate (Denks et al., 2014). Flavivirus polypeptide insertion into the ER membrane is thus at least partially reliant on the interaction with SRP and SEC proteins. Unsurprisingly, these proteins have been found in several flavivirus-host protein interaction studies, including interactions between ZIKV/DENV NS4A with SEC62, SEC61γ, and SRPR, NS4A/2B with SEC61β, and NS4B with SEC61α (Scaturro et al., 2018; Shah et al., 2018). Along with these interaction-based screens, these proteins have been identified in genetic screens as host factors supporting flavivirus replication (Krishnan et al., 2008; Sessions et al., 2009; Marceau et al., 2016; Zhang et al., 2016; Hoffmann et al., 2021). Interestingly, while the SEC61 translocon is essential for protein biogenesis, pharmacological modulation of this complex inhibits DENV and ZIKV replication (Heaton et al., 2016; Monel et al., 2017; Shah et al., 2018). Together, these results highlight the importance of the SRP/SEC61 translocon on flavivirus replication and the potential for a pan-flaviviral drug target.

### ER Membrane Complex

The EMC co-translationally interacts with nascent proteins and prevents their degradation by associating with chaperones. The EMC preferentially stabilizes multipass membrane proteins that may otherwise have difficulty being inserted into the ER membrane, thereby avoiding misfolding and degradation (Jonikas et al., 2009; Shurtleff et al., 2018). Similar to the SEC61 complex, EMC proteins have been identified in a number of flavivirus protein interaction (Coyaud et al., 2018; Shah et al., 2018) and genetic screens (Ma et al., 2015; Marceau et al., 2016; Savidis et al., 2016; Hoffmann et al., 2021), underlining their importance. Three recent papers dissect their role in flavivirus replication. Through a combination of biochemical assays and pulse-chase experiments with gene knockdown, Lin et al. (2019) demonstrated that EMC1 promoted NS4B biogenesis, but not its post-translational stability. Interestingly, NS4B’s dependence on the EMC arises from its two N-terminal transmembrane regions, which are marginally hydrophobic, as altering the nature of these regions in either direction, more or less hydrophobic, rescued expression in EMC knockout cells. Together, this suggests that the generation and co-translational stability of flavivirus multi-pass proteins, including NS4B, depends on the interaction with EMC for protection from degradation and integration into the membrane (Lin et al., 2019). Similar inhibition of virus replication and decreases in viral protein production were also shown by (Ngo et al., 2019), using a similar CRISPR knockout setup. Using a dual-fluorescence reporter system they were able to identify that NS4B’s underlying reliance on the EMC stems from its link to NS4A through the 2 K peptide, a transmembrane region which serves as a signal sequence for the translocation of NS4B (Ngo et al., 2019). Barrows et al. (2019) additionally found that knockout of EMC4 led to near complete loss in replication of DENV, ZIKV, and YFV, but did not affect WNV replication at all. They speculated this difference may arise from the WNV being transmitted by *Culex* mosquitoes, rather than *Aedes*. This vector specific hypothesis was supported by their finding that DENV titer in *Aedes* mosquito midguts was reduced after siRNA targeting of EMC2/3/4, although the decreases in replication were not nearly as severe as what was observed in human cells (Barrows et al., 2019). Altogether, the EMC is a vital host factor utilized by *Aedes*-transmitted flaviviruses for correct viral protein insertion into the ER membrane.

### Signal Peptidase and Oligosaccharyltransferase Complexes

Gene perturbation screens have long been used to identify essential flavivirus host factors. With the advances of CRISPR, these screens have become even more powerful. A pair of such screens published together in 2016 mapped host factors of multiple flaviviruses (Marceau et al., 2016; Zhang et al., 2016). Unsurprisingly, proteins involved in ER translocation of and polypeptide stability, including SEC61B and EMC proteins, were among the hits in these screens. Additionally, signal peptidase complex (SPCS) and oligosaccharyltransferase (OST) complex were found to be essential for replication of many flaviviruses. Knockout of SPCS1, a major component of the SPCS, in HEK293T embryonic kidney cells completely ablated replication of all tested flaviviruses, but had little effect on other unrelated RNA viruses, suggesting its role in virus replication was specific to *Flaviviridae*. Further experiments revealed that the SPCS1 is responsible for several polyprotein cleavage events, specifically C-prM, prM-E, E-NS1, and 2 K-NS4B (Zhang et al., 2016). The cleavage between NS1 and NS2A occurs through an unknown signal peptidase pathway mechanism. Interestingly, the OST complex plays a role in replication separate from its enzymatic activity. Normally, the OST complex is responsible for the *N-*linked glycosylation of host proteins. Knockout of major OST complex component STT3A had major effects on the replication of DENV, YFV, WNV, JEV, and ZIKV (Marceau et al., 2016; Zhang et al., 2016). However, these replication defects could be rescued by the expression of catalytically dead STT3A mutants, suggesting that the OST complex serves virus replication through function outside its ability to glycosylate proteins (Marceau et al., 2016). Physical interactions with flavivirus replication complex members NS1, NS2B, NS3, and NS4B along with its close association with sites of virus replication in the ER suggest that the OST complex may serve a structural role in genome replication (Marceau et al., 2016; Hafirassou et al., 2017).

## ER Remodeling and Virus Replication Compartment Formation

The majority of flavivirus replication occurs within the ER membrane. Flaviviruses employ a variety of mechanisms to remodel the host ER into a niche, which maximizes the efficiency of genome replication and viral packaging. The task of remodeling the ER is primarily performed by the viral NS proteins through a combination of direct remodeling and specific virus-host interactions. NS4A and NS4B contain transmembrane domains, which pass through the ER and helices that lie in the plane of the ER lumen to induce positive curvature of the membrane (Roosendaal et al., 2006; Miller et al., 2007; Kaufusi et al., 2014). Membrane alteration is further driven by the oligomerization of NS4A (Lee et al., 2015). In addition to the action of these viral proteins on their own, they recruit and hijack the function of a number of other host proteins involved in ER morphology. The highly curved and tubular host ER membrane system is stabilized and maintained by several protein families including the reticulon (RTN) family, the atlastin (ATL) family, and the Lunapark (LNP) protein (Goyal and Blackstone, 2013; Wang et al., 2016). Even beyond these canonically ER proteins, other host factors have been shown to be involved in flavivirus-mediated ER modifications. Recently several groups have evaluated the roles of these protein families in flavivirus replication and ER remodeling during infection.

### Reticulon

Proteins in the RTN family all have a domain including two transmembrane regions separated by a single hydrophilic loop which, similarly to NS4A, induce membrane curvature (Zurek et al., 2011). Reticulon 3A (RTN3A) is known to be involved in the replication of other viruses, including the *Flaviviridae* family member Hepatitis C virus (HCV; Tang et al., 2007; Diaz et al., 2010; Barajas et al., 2014). Aktepe et al. (2017) found that the broadly expressed RTN3.1A plays a role in the replication of several flaviviruses, including WNV, DENV, and ZIKV. RTN3.1A colocalized with sites of virus replication and siRNA silencing resulted in significant decreases in viral titer after infection. ZIKV infection in RTN3.1A-silenced cells displayed dramatically less membrane curvature with fewer replication complexes. Using a combination of immunofluorescence microscopy and fluorescence resonance energy transfer (FRET), the authors determined that RTN3.1A specifically interacted with WNV NS4A, whereas ZIKV and DENV NS4A did not (Aktepe et al., 2017). However, later proteomics studies did identify an interaction between ZIKV NS4A and RTN3 (Shah et al., 2018). A yeast-two hybrid screen also showed that ZIKV NS4A and NS2B interact with RTN1, suggesting that flaviviruses may utilize RTN family members differentially for roles in ER remodeling (Golubeva et al., 2020).

### Atlastins

The ATL family of proteins is composed of three (ATL1/2/3) membrane-bound, dynamin-related GTPases that function in maintaining Golgi (ATL1) and ER (ATL2/3) morphogenesis through the formation of three-way junctions (Rismanchi et al., 2008; Hu et al., 2009). ZIKV is known to actively remodel the ER and induce the formation of large ER-derived cytoplasmic vacuoles. Ultimately this results in cell death through paraptosis, a caspase-independent, non-apoptotic form of cell death (Monel et al., 2017). The formation of these vacuoles in HeLa cells is dependent on the activity of ATLs. Knockout of ATL2 and ATL3 led to nearly a complete loss in the formation of these vacuoles during ZIKV infection and significant reduction in ZIKV replication. These phenotypes could be rescued by expression of wild-type ATL3, but not a GTPase-deficient mutant (Monel et al., 2019). Another group similarly found that knockdown of ATL2/3 reduced the replication of both ZIKV and DENV and that ATL3 played an important role in DENV maturation (Neufeldt et al., 2019). There appears to be multiple methods by which flaviviruses physically interact with ATL proteins. Using co-immunoprecipitation (Co-IP) and immunofluorescence analysis, one group determined that ATL3 strongly interacted with both ZIKV NS2A and NS2B3, although they did identify partial interaction with both NS4A and NS4B as well (Monel et al., 2019). ATL2/3 was found to interact with DENV NS2B, NS3, and NS5. Interestingly, ATL3 was also found to further interact with DENV NS1, envelope, and capsid proteins (Neufeldt et al., 2019). Flavivirus-ATL interactions have also been identified in a number of proteomic screens including WNV NS4B with ATL2 (Li et al., 2019) and ZIKV NS4A and NS2A with both ATL1 and ATL2 (Coyaud et al., 2018). Thus, while multiple flaviviruses hijack atlastin proteins, the molecular mechanisms appear to be unique.

### Lunapark

While RTN family proteins induce curvature within the ER membrane and ATL form three-way tubular junction, the LNP protein stabilizes these junctions and is required for their mobility, a necessary feature of the dynamic ER (Chen et al., 2015). Similar to RTN and ATL, siRNA silencing of LNP results in significant reduction in flavivirus induced replication compartments and corresponding decreases in genome replication. Using Co-IP, Tran et al. (2021) identified that TBEV NS4B interacted with LNP through its C-terminal region (Tran et al., 2021). Additionally, ZIKV NS4A has been identified to interact with LNP, and may constitute virus-specific mechanisms of ER-remodeling (Shah et al., 2018). All together, these findings show that flaviviruses physically hijack a number of host pathways to remodel the ER membrane system to create a space conducive to virus replication.

### TMEM41B and VMP1

A recent CRISPR genetic screen assessed host factors involved in flavivirus infection (Hoffmann et al., 2021). In addition to identifying many ER proteins discussed previously, the authors also identified two transmembrane ER proteins, TMEM41B and VMP1. These proteins function as phospholipid scramblases and have similar roles in lipid mobilization, lipoprotein biogenesis, autophagy, and the induction of membrane curvature (Zhao et al., 2017; Moretti et al., 2018; Morita et al., 2019; Huang et al., 2021). Knockout of either gene dramatically inhibited the replication of a wide range of mosquito- and tick-borne flaviviruses. TMEM41B was also shown to be critical for infection across multiple cell types, including mosquito C6/36 cells. The authors found TMEM41B interacts and colocalizes with ZIKV NS4A and YFV NS4B during infection, which is supported by the previous identification of ZIKV NS4B’s interaction with TMEM41B (Scaturro et al., 2018). Given its role in inducing membrane curvature, this suggested that TMEM41B may be involved in the formation of viral replication compartments in the ER. Intriguingly, TMEM41B-deficient cells were observed to have heightened innate immune responses after infection. Using YFV replicons, this was elegantly shown to be due to increased sensing of viral dsRNA in TMEM41B knockout cells (Hoffmann et al., 2021). Together, these results show that TMEM41B is a pan-flavivirus host factor that is likely involved in the formation of replication compartments in the ER, and loss of this protein results in the inability to retain viral dsRNA in the ER, leading to detection by host immune response sensors.

### Vimentin

The RTN, ATL, and LNP family proteins are logical targets for virus-mediated ER remodeling based on their canonical roles in regulating ER morphology. However, flaviviruses are also capable of hijacking host proteins with more divergent functions to establish replication compartments within the ER. A 2014 study by Teo and Chu (2014) established that DENV NS4A interacted with host vimentin, a major component of cytoskeletal intermediate filaments, to anchor replication compartments in the ER. They found that the N-terminal cytoplasmic region of NS4A mediated this interaction and that DENV infection increasing the phosphorylation of vimentin, promoting depolymerization and reassembly to the perinuclear region where it was utilized for virus replication. Phosphorylation of vimentin was shown to be crucial for replication as siRNA silencing of the vimentin-targeting kinase CaMKIIγ led to significant decreases in DENV replication (Teo and Chu, 2014).

### Receptor for Activated C Kinase 1

The Receptor for Activated C Kinase 1 (RACK1) protein is a known scaffolding protein with roles in protein shuttling, anchoring, and stabilization, as well as mediating cellular pathways through protein interactions (Adams et al., 2011). The interaction between DENV NS1 and host RACK1 was first identified in a DENV NS1 specific proteomics screen (Hafirassou et al., 2017) but the role of this interaction was not fully explored until recently by Shue et al. (2021). They performed a genome-wide CRISPR knockout screen in Huh7 cells to identify host genes involved in ZIKV replication. This identified several potential host genes including members of the EMC (discussed earlier in this review), as well as RACK1. Additionally, they found that silencing of RACK1 impacted the replication of several flaviviruses, including ZIKV, DENV, WNV, POWV, and LGTV, and even SARS-CoV-2. However, they found that YFV, herpes simplex virus (a DNA virus), and vesicular stomatitis virus (ssRNA- virus) were not affected by RACK1 silencing. Using a *Renilla luciferase* DENV replicon they determined that RACK1 specifically played a role in viral genome replication, rather than viral entry or translation. Using replication-independent expression system that induces the formation of replication compartments in the ER without virus infection they found that RACK1 silencing led to reduced formation of these compartments in the ER (Shue et al., 2021). These studies are a great example of the power of integrating proteomic and genetic screens to identify mechanisms of virus replication. In the future utilization of existing screens will advance our understanding of these mechanisms and identify new interactions that are necessary for flavivirus replication.

One interesting feature of flavivirus infection worth noting is the induction of convoluted membranes (Figure 3). These peculiar membranous structures contain vast arrangements of smooth ER, however, they appear to form only under certain conditions, as their presence can vary with virus or cell type (Junjhon et al., 2014; Hanners et al., 2016; Cortese et al., 2017; Offerdahl et al., 2017). Convoluted membranes contain viral proteins but lack viral RNA, suggesting these are not sites of genome replication (Welsch et al., 2009). The virus-host PPIs that contribute to the formation of these membrane structures are still under investigation. It has been shown that NS4B associated with mitochondria physically contact these structures, potentially to tether them near sites of virus replication or assembly or to dampen innate immune response signaling (Chatel-Chaix et al., 2016).

## Flavivirus Interactions for Host Processes Outside the ER

While the ER is a major site of flavivirus replication, virus-host protein interactions in other organelles are critical for replication. Soluble viral proteins such as NS3 and NS5 are known to have dispersed localizations during infection, thus it is unsurprising that identified interacting host proteins also have a wide range of localizations. Here, we will review important and recently identified virus-host PPIs outside context of ER replication that promotes virus replication.

### Trafficking

After virions are packaged and assembled in the ER lumen they must be processed prior to release. Specifically, the prM protein on the outermost part of the virion must be cleaved by furin, a host protease within the Golgi apparatus. Cleavage sites on prM are only made accessible by the relatively acidic environment of the Golgi and secretory vesicles (Stadler et al., 1997; Yu et al., 2008; Zheng et al., 2014). This prM maturation is required to allow future viral entry into host cells after release. Vesicles containing immature virions reach the Golgi through the host’s secretory pathway or trans-Golgi network (TGbN). Golgi proteins and others involved in TGN trafficking have been identified in proteomic screens (Carpp et al., 2014), but in-depth studies on the role of these interactions in flavivirus replication are very limited. Recently several specific virus-host PPIs have been identified here with roles in virus maturation and replication.

As trafficking through the TGN is essential for flaviviruses, one anti-viral host mechanism is to limit this processing by halting virion progression at the Golgi, preventing release. One well-studied protein with this function is bone marrow stromal cell antigen 2 (BST2), also known as tetherin. BST2 is known to restrict the replication of many viruses, including filoviruses, retroviruses, and alphaviruses, by tethering virions to the cell surface or by interrupting virion release from the TGN prior to exit from the cell (Jouvenet et al., 2009; Sakuma et al., 2009; Liu et al., 2015; Ooi et al., 2015). Accordingly, several viruses have evolved measures to counteract this inhibition. For example, the Vpu protein of HIV-1 inhibits the anti-viral tethering effects of BST2, allowing release of infectious virions from the cell (Neil et al., 2008). However, there are some conflicting reports about the effects of BST2 on flaviviruses. A 2012 study described significant BST2-mediated inhibition of DENV release from Huh7 cells (Pan et al., 2012). Conversely, another study found only modest effects on non-infectious, “virus-like particle” release from TRex HEK293 cells expressing BST2 and transfected to express DENV Env (Ooi et al., 2015). Whether these discrepancies are methodological or cell-type derived is unclear. More recently, Li et al. (2017) investigated the potential mechanisms by which JEV escapes BST2 restriction. Endogenous BST2 proteins levels were actively decreased during JEV infection and expression of JEV Env alone was sufficient to reduce BST2 expression. JEV Env physically interacted with BST2 at its transmembrane and cytoplasmic loop domains, and targeted it for lysosomal degradation (Li et al., 2017). Thus, the interaction between Env and BST2 promotes virus replication by eliminating the anti-viral activity of BST2. Whether other flaviviruses interact with and inhibit BST2 using similar mechanisms requires further study. Previously we also discussed the interaction between ATL3 and multiple viral proteins, and the role of this interaction in ER remodeling. Interestingly, this study also identified a role of ATL3 in flavivirus maturation and furin recycling. Knockdown of ATL3 increased levels of extracellular un-cleaved prM and altered furin localization away from the Golgi. The relocalization of furin was specifically observed after knockdown of ATL2 and ATL3, whereas knockdown of other ER remodeling proteins RTN3 and LNP had no effect (Neufeldt et al., 2019).

### Autophagy

Autophagy is an essential intracellular degradative process that recycles cytoplasmic components (Bento et al., 2016). Autophagy involves three major steps, the formation of autophagosomes and simultaneous capture of cytoplasmic material, the fusion of autophagosomes with lysosomes to form autolysosomes, and the turnover of autolysosomes. The cytoplasmic components often referred to as cargo, can either be selectively or non-selectively degraded (Gatica et al., 2018). For selective autophagy, cargo such as mitochondria is tagged by cargo receptors which are then encapsulated and degraded by autophagy. Autophagy is involved in the replication of various flaviviruses. The overall role of autophagy as proviral or antiviral in flavivirus replication is complex and has no clear consensus (Choi et al., 2018; Ke, 2018; Echavarria-Consuegra et al., 2019). Here, we discuss the studies that have implicated the role of autophagy-related proteins in virus replication through physical interactions with viral proteins.

DENV and ZIKV hijack various aspects of selective autophagy for efficient virus replication. Regulation of lipid metabolism during DENV infection has been reported by multiple groups (Heaton and Randall, 2010; Perera et al., 2012; Jordan and Randall, 2017; Chotiwan et al., 2018; Koh et al., 2020). DENV NS4A physically interacts with unubiquitinated AUP1 to translocate lipid droplets to autophagosomes to induce lipophagy (Zhang et al., 2018). Interestingly, DENV NS4B or DENV infection is essential for this interaction. Ubiquitination of AUP1 impeded its interaction with NS4A, which led to defective lipophagy and reduced viral titers. This study highlights the importance of lipophagy during virus infection that is regulated by virus-host protein interactions. Regulation of apoptosis through autophagy is another strategy utilized by DENV for prolonged virus replication (McLean et al., 2011), and DENV NS1 interacts with Beclin-1 to activate autophagy and prevent apoptosis at early stages of infection (Lu et al., 2020). FAM134B, an ER phagy (reticulophagy) selective cargo receptor was identified to interact with DENV and ZIKV NS2B3 (Lennemann and Coyne, 2017). The researchers demonstrated that DENV and ZIKV NS2B3 cleave FAM134B to inhibit the degradation of viral proteins through reticulophagy. Additionally, overexpression of FAM134B leads to decreased virus replication. These results indicate selective degradation of ER is subverted by viruses even though overall autophagy could be upregulated during infection. In a recent study, Ponia and colleagues observed inhibition of mitophagy through the interaction of ZIKV NS5 with the host protein Ajuba (Ponia et al., 2021). Ajuba is a key regulator of mitophagy and is translocated to depolarized mitochondria to initiate PINK1-Parkin mediated mitophagy. NS5 interaction with Ajuba impeded its translocation to depolarize mitochondria, thus inhibiting mitophagy. The authors further use *in vivo* ZIKV infection studies in mice to demonstrate increased early pro-inflammatory chemokines and viral load in tissue due to inhibition of mitophagy, further underlining the importance of the NS5-Ajuba interaction. These studies point towards the regulation of selective autophagy by DENV and ZIKV. Systematic measurements of degraded cargo during virus infection can provide key insights. In the future, it will also be valuable to explore if modulation of selective autophagy is a common theme for other flaviviruses and other types of selective autophagy (e.g., pexophagy and xenophagy).

Interactions of general autophagy-related proteins with WNV and JEV proteins have also been identified. WNV Capsid protein interacts with AMPK, an autophagy inducer (Kobayashi et al., 2020). This interaction mediated the degradation of AMPK through the proteasome pathway and led to the accumulation of ubiquitinated protein aggregates. A mutant Capsid protein reduced the interaction with AMPK and its degradation. Even though disrupting this interaction did not affect virus replication, it led to lower protein aggregates in mouse brain and reduced neurological symptoms. For JEV, Sharma et al. (2021) have shown that autophagy acts as an antiviral response during JEV infection in neuronal cells. They also observed NS1 colocalization with LC3-I, an important autophagy protein whose depletion caused decreased viral titers. In a more recent study, the same group also demonstrated Capsid protein interaction with LC3-I, using immunoprecipitation (Sarkar et al., 2021). The functional role of these interactions in virus replication and autophagy is uncharacterized and could be a potential study.

Future efforts can be focused on investigating known uncharacterized physical interactions. The mTOR pathway is an important autophagy pathway that is differentially regulated during flavivirus infection (Shives et al., 2014; Liang et al., 2016; Jordan and Randall, 2017; Sahoo et al., 2020; Lahon et al., 2021). Moreover, viral protein interactions with mTOR were also found using proteomic approaches (Shah et al., 2018). However, characterizing the role of virus-mTOR PPIs in the context of virus infection is largely unexplored and could be a potential future direction. Selective autophagy during virus infection is another interesting attribute for potential study. Viruses appear to exploit the selective nature of autophagy by variably regulating specific cargo degradation. For example, DENV upregulates lipophagy while downregulating reticulophagy for effective replication (Lennemann and Coyne, 2017; Zhang et al., 2018). Thus, further investigating the interactions found between the viral proteins and cargo receptors could be a promising direction. We explored the literature to generate a list of virus-autophagy PPIs that have been identified in various proteomic screens, which could further explain the role of autophagy during virus infection (Table 1). Interestingly, while many autophagy proteins were identified in these screens, none were pursued mechanistically in those published studies, leaving the door open to many systematic studies of these PPIs. Finally, capturing the temporal change in cargo degraded during virus infection may also provide novel insights into the dynamic replication cycle.

**Table 1:** Protein–protein interaction (PPI) found between autophagy proteins and viral proteins from seven data sets (Coyaud et al., 2018; Scaturro et al., 2018; Shah et al., 2018; Li et al., 2019; Golubeva et al., 2020; Zeng et al., 2020).

Autophagy protein	Autophagy related role	Viral proteins
ACBD5	Pexophagy receptor	NS4A^1^
AMBRA1	Key regulator of autophagy by modulating the BECN1-PIK3C3 complex	NS1^7^, NS2B^7^
ATG9A	Supplies membrane for the growing autophagosome	Env^7^
BNIP3 (NIP3)	Mitophagy receptor	NS5^1^^,^^2^
EI24 (EPG4)	Regulates formation of degradative autolysosomes	NS1^2^, NS4B^3^
LGALS8	Restricts infection by initiating autophagy *via* interaction with CALCOCO2/NDP52	NS3^6^
MTOR	Key regulator of autophagy through phosphorylation of ULK1, DAP, AMBRA1, and RUBCNL	NS4A^1^^,^^2^
PHB2	Mitophagy receptor	NS2B3^3^, NS4B^3^
SQSTM1 (p62)	Multiple cargo receptor	NS4B^2^
STX17	Regulates autophagosome fusion with lysosomes	NS2A^7^
VCP	Essential for the maturation of ubiquitin-containing autophagosomes and the clearance of ubiquitinated protein by autophagy	NS2B3^5^
WAC	Regulator of autophagy	NS2B^6^
AUP1	Lipophagy regulator	NS2A^4^, NS4B^3^^,^^4^
FAM134C	Reticulophagy receptor	NS4A^1^, NS4B^3^
RTN3	Reticulophagy receptor	NS4A^1^
SEC62	Reticulophagy receptor	NS4A^2^
CALCOCO1	Reticulophagy receptor	NS5^5^
NBR1	Aggrephagy, pexophagy, and xenophagy receptor	NS2A^4^
VMP1	Required for autophagosome biogenesis	NS4A^4^
TMEM41B	Required for autophagosome biogenesis	NS4B^3^

1 Shah et al. (2018) (ZIKV).

2 Shah et al. (2018) (DENV).

3 Scaturro et al. (2018) (ZIKV),

4 Coyaud et al. (2018) (ZIKV).

5 Li et al. (2019) (WNV).

6 Golubeva et al. (2020) (ZIKV).

7 Zeng et al. (2020) (ZIKV).

PPI that were found significant by the authors were considered for the search. Approximately, 100 autophagy proteins were probed for interactions based on a list of proteins mentioned in these studies (Galluzzi et al., 2017; Gatica et al., 2018; Gubas and Dikic, 2021).

### Mitochondrial Dynamics and Morphology

Mitochondria are dynamic organelles with widespread functions in cellular homeostasis, including ATP production, immune response signaling, and apoptosis activation. Unsurprisingly, many viruses interact with and perturb these functions to benefit their own replication (Anand and Tikoo, 2013). Recently the mechanisms and protein interactions that flaviviruses use to modulate these mitochondrial functions have revealed dynamic alterations in mitochondrial morphology that impact virus replication.

The morphology of host mitochondria is constantly changing. The constant fusion and fission of mitochondria is critical for cellular homeostasis. The fusion of mitochondria together is mediated by mitofusin 1 (MFN1) and MFN2 in the outer mitochondrial membrane and optic atrophy protein 1 (OPA1) in the inner membrane. Fission is mediated by dynamin-related protein 1 (DRP1), which is soluble and recruited to mitochondria by mitochondrial fission protein 1 (FIS1; Westermann, 2010). ZIKV and DENV both impact mitochondrial morphology, albeit in cell-type and virus specific manners (Chatel-Chaix et al., 2016; Barbier et al., 2017; García et al., 2020; Yang et al., 2020). DENV and ZIKV infection in Huh7 hepatocytes induces dramatic mitochondrial elongation. This is associated with significant decreases in DRP1 fission activity, specifically through decreased phosphorylation at S616, a site which induces fission by DRP1. NS4B interacts with many mitochondrial proteins (Scaturro et al., 2018; Shah et al., 2018) and its expression alone is sufficient to alter mitochondrial morphology (Chatel-Chaix et al., 2016). NS4B expression is linked with decreased expression of CDK1, the kinase which phosphorylates DRP1 at S616. Knockdown of DRP1 further increases DENV and ZIKV replication, while increasing fusion through knockdown of MFN2 decreases replication (Chatel-Chaix et al., 2016; Barbier et al., 2017). Interestingly, knockdown of DRP1 did not impact the replication of fellow flavivirus WNV or the closely related HCV (Chatel-Chaix et al., 2016). Together, this suggests that DENV and ZIKV specifically induce the elongation of mitochondria in these cells. It appears this elongation may serve two functions for virus replication. Firstly, elongated mitochondria have increased respiratory function, resulting in greater energy production which may be utilized directly for virus replication or promote host cell survival (Barbier et al., 2017). Secondly, this elongation impedes mitochondrial innate immune response signaling by preventing the translocation of RIG-I to mitochondrial-associated membranes and decreasing mitochondrial antiviral signaling protein (MAVS)-associated interferon (IFN) production (Yu et al., 2015; Chatel-Chaix et al., 2016). The interplay between flaviviruses and innate immune signaling, including through MAVS and RIG-I, will be discussed in more detail later in this review.

Intriguingly, mechanisms that promote mitochondrial fission, rather than fusion, have been observed during DENV and ZIKV infection in other cell types (Yu et al., 2015; Yang et al., 2020). In A549 cells, DENV infection also leads to abnormal mitochondrial dynamics, however, independent of DRP1. Rather, MFN1 and MFN2 are cleaved by the DENV NS2B3 protease, resulting in decreased fusion and more mitochondrial fragmentation. Specifically, cleavage of MFN1 results in decreased MAVS-mediated IFN production, while cleavage of MFN2 decreased the activation of cell-death associated caspases. Again, this activity does not appear to be conserved across all flaviviruses, as NS2B3 from JEV was unable to perform the same cleavage events (Yu et al., 2015). This specificity may contribute to the unique pathogenesis of some flaviviruses. Congenital ZIKV infection is associated with the development of neurological and ocular abnormalities, which are not observed with other flaviviruses (Roach and Alcendor, 2017; Carod-Artal, 2018). It is possible that perturbation of mitochondrial processes by viruses are especially potent in these tissues, as metabolic demands are high and these tissues are very sensitive to mitochondrial dysfunction (Pacheu-Grau et al., 2018; Norat et al., 2020) In ZIKV-infected neural stem cells (NSCs) mitochondria numbers and size are significantly decreased, associated with concomitant decreases in MFN2 protein expression, whereas the other fusion/fission proteins (MFN1, OPA1, DRP1, and FIS1) were unchanged (Yang et al., 2020). ZIKV had similar effects on the mitochondria of retinal pigment epithelial (RPE) cells, with mitochondria appearing more fragmented and punctate in nature (García et al., 2020). In both cases, ZIKV-associated morphology changes involved the loss of mitochondrial membrane potential, resulting in diminished ATP production and mitochondrial function. Whether ZIKV NS2B3 performs similar cleavage of MFN2 as the DENV protease or if ZIKV relies on other unique interactions requires further experimentation.

## Antagonism of Host Immunity by Flavivirus-Host PPIs

**Figure 4:**
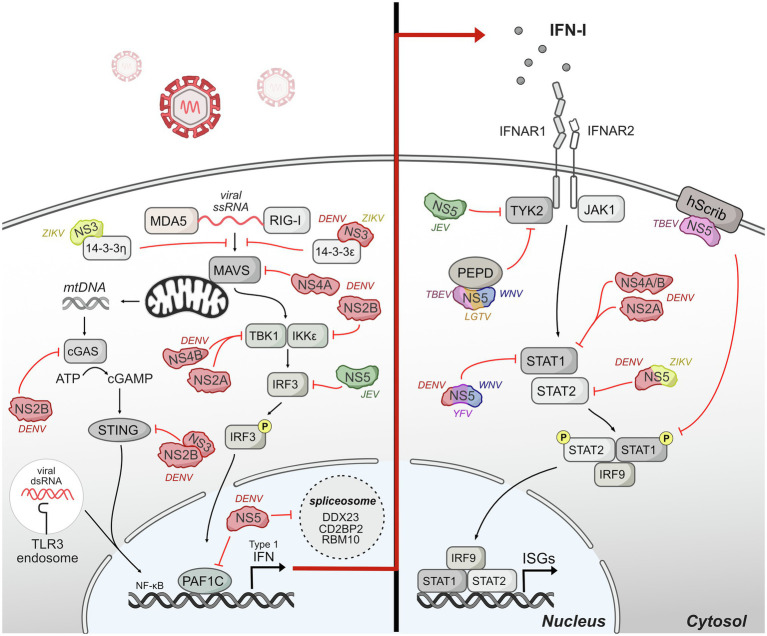
Host innate immune response is antagonized by flavivirus protein interactions. Upon entry, flaviviruses are sensed by different pattern-recognition receptors (PRRs) such as TLR-3 and RIG-I. The signaling induced by these sensors converges on a common cascade that induces the production of interferon (IFN) and downstream genes stimulated by IFN called interferon-stimulated genes (ISGs). Flaviviruses have evolved invasive strategies to interfere with host immune response by antagonizing different protein components of innate immune signaling pathways associated with IFN production and IFN signaling. The DENV NS2B3 protease is shown as NS2B and NS3 interacting together to antagonize STING.

While some interactions between viral proteins and host proteins associated with the immune system restrict flavivirus replication and pathogenesis (Sun et al., 2013a; Douradinha et al., 2014; Wessel et al., 2021), flaviviruses have evolved numerous mechanisms to sabotage the host innate immune response *via* interactions with host proteins. Here, we review the major mechanisms of antagonism associated with IFN production, IFN signaling, and the complement system (Figure 4).

### IFN Production

Pattern-recognition receptors (PRRs) sense flaviviruses upon entry. The major PRRs relevant for flaviviruses are TLR3 and TLR7/8, which are located primarily in endosomal vesicles and recognize viral RNA of incoming virions (Heil et al., 2004; Tsai et al., 2009); retinoic-acid inducible gene-I (RIG-I), and myeloma differentiation factor 5 (MDA5), which recognize cytosolic RNA (Yoneyama et al., 2005); and cyclic GMP-AMP synthase (cGAS) which recognize cytosolic DNA (Sun et al., 2013b). Mechanistically, activated TLR3 and TLR7 recruited adaptor protein MyD88 and TRIF to initiate further changes regulating the expression of cytokines, chemokines, and type I IFNs (Kawasaki and Kawai, 2014). After sensing viral RNA, RIG-I and MDA5 move from the cytosol to mitochondria and interact with their adaptor, mitochondrial antiviral signaling protein (MAVS), to continue further downstream signaling that activates IRF3 and NF-kβ (Chiang et al., 2014). cGAS activation after recognizing cytosolic DNA catalyzes the synthesis of cyclic GAMP (cGAMP) which activates STING, which subsequently activates IFN expression (Cai et al., 2014). The signaling induced by these sensors converges on a common cascade that induces the production of IFN and downstream genes stimulated by IFN called interferon-stimulated genes (ISGs; Nasirudeen et al., 2011).

Flaviviruses have evolved different strategies to interfere with the host production of IFNs. A major mechanism for flaviviruses is to disrupt double-stranded RNA-sensing pathway. NS3 from DENV and ZIKV binds 14-3-3ɛ, an important protein in antiviral immunity (Liu et al., 2012), *via* a conserved phosphomimetic motif on NS3 and prevent the translocation of RIG-I to mitochondria, and consequently IFN production (Chan and Gack, 2016; Riedl et al., 2019). During DENV infection, DENV NS4A also physically interacts with MAVS to prevent RIG-I from forming complexes with MAVS in mitochondria-associated endoplasmic reticulum membranes (MAMs), leading to the disruption of RIG-I-induced IRF3 activation and subsequently suppression of IFN production (He et al., 2016).

Further down in the RIG-I-induced type I IFN pathway, DENV serotypes 1, 2, and 4 (DENV1, DENV2, and DENV4) NS2A and NS4B proteins inhibit RIG-I/MDA5-regulated interferon beta (IFN-β) induction by blocking TBK1/IRF3 (Dalrymple et al., 2015). DENV NS2B3 interacts with IKKe to prevent IRF3 phosphorylation (Angleró-Rodríguez et al., 2014) potentially changing the activation of multiple antiviral genes including type I IFN. JEV inhibits IFN-β production by suppressing IFR3 and NF-kβ (Ye et al., 2017). Mechanistically, JEV NS5 interacts with nuclear transport proteins KPN2, KPN3, and KPN4 and blocks their interaction with IRF3 and P65, therefore preventing nuclear translocation of IRF3 and NF-kβ and reducing type I IFN production.

Even as RNA viruses, flaviviruses also antagonize IFN production by interfering with cytosolic DNA-sensing pathway by cGAS and its adaptor STING. Specifically, as DENV infection triggers innate immune response through mtDNA sensing by the DNA sensor cGAS (Sun et al., 2017), DENV NS2B targets cGAS for degradation to prevent the detection of mitochondrial DNA released during DENV infection, blocking the activation of cGAS/STING pathway and the induction of type I IFN (Aguirre et al., 2017). DENV NS2B3 also physically interacts with and cleaves STING to inhibit type I IFN production in species-specific manner (Aguirre et al., 2012; Yu et al., 2012). During ZIKV infection, cGAS is targeted and cleaved by NS1-stablized caspase-1, leading to enhanced NLRP3 inflammation activation and reduced type I induction to benefit the infection (Zheng et al., 2018). The multiple mechanisms by which flaviviruses antagonize DNA sensing suggest that this is an important mechanism of immune evasion for RNA viruses and represents a new frontier of investigation for virus-host interactions.

Flavivirus NS5 proteins also antagonize innate immunity upstream of IFN production and signaling, primarily through interactions with host gene expression machinery in the nucleus. The extent of NS5 localization to the nucleus varies depending on flavivirus species, yet some nuclear localization and nuclear/cytoplasmic shuttling appears to occur for nearly all NS5s, with the exception of duck Tembusu virus (Hannemann et al., 2013; Zhao et al., 2015; Grant et al., 2016; Kumar et al., 2016; Tay et al., 2016; Duan et al., 2019; Cheng et al., 2021; Petit et al., 2021). NS5 protein interactions with host gene expression machinery have been noted through several unbiased screens, including mass spectrometry and yeast-two-hybrid screens (Khadka et al., 2011; Le Breton et al., 2011; De Maio et al., 2016; Coyaud et al., 2018; Scaturro et al., 2018; Shah et al., 2018; Li et al., 2019). Several of these protein interactions have been linked back to NS5 perturbation of host gene expression. For example, a proteomic study of DENV NS5 during infection revealed interactions with CD2BP2 and DDX23, core components of U5 small nuclear ribonucleoprotein particles (U5 snRNPs) that ultimately interfere splicing efficiency (De Maio et al., 2016). DENV NS5 also dysregulates host splicing by physically interacting with RBM10, a splicing factor that regulates spermidine/spermine-N1-acetyltransferase (SAT1) splicing and promoting RBM10 proteasomal degradation. The interaction potentially restricts RBM10 from its proinflammatory function and benefits DENV replication (Pozzi et al., 2020). In our studies on DENV-human protein interactions, we identified an interaction with PAF1C (Shah et al., 2018), which regulates the transcription elongation of many immune response genes (Marazzi et al., 2012; Parnas et al., 2015). Our recent work dissecting the NS5-PAF1C interaction demonstrated that PAF1C regulates immune response genes upstream of type I IFN production, including the RIG-I/DDX58 signaling axis. Breaking the NS5-PAF1C interaction through mutagenesis of NS5 rescued PAF1-dependent gene expression, underlining the importance of this protein interaction (Petit et al., 2021).

The recurring theme of NS5 nuclear localization and interactions with nuclear proteins has led to much speculation in the field regarding why the polymerase and methyltransferase of a cytoplasmic RNA virus would have such behavior. There is mounting evidence that flavivirus NS5 protein can perturb host gene expression, both through dissection of virus-host protein interactions, and through more generalized gene expression studies. For example, independent studies of ZIKV, WNV, and DENV NS5 all point to overall inhibition of immune gene expression (López-Denman et al., 2021; Petit et al., 2021; Zhao et al., 2021). On the other hand, studies involving infection have not revealed *in vitro* or *in vivo* phenotypes for DENV mutants that reduce NS5 nuclear localization (Kumar et al., 2016; Tay et al., 2016; Cheng et al., 2021), resulting in skepticism regarding the biological significance of NS5 nuclear localization. However, for studies involving DENV serotype 2, it should be noted that there are two distinct nuclear localization signals (NLSs) that contribute to nuclear localization. In fact, mutation of a single NLS still results in substantial NS5 nuclear localization (~1:1 nuclear:cytoplasmic ratio). In our own studies, we show that only mutation of both NLSs truly excludes NS5 from the nucleus and disrupts the NS5-PAF1C interaction (Petit et al., 2021). Thus, a modest amount of NS5 nuclear localization may be sufficient for its role in perturbing host gene expression. Using a double NLS mutant to study protein interactions and virus replication phenotypes will be essential to understanding the true function of nuclear NS5. Creating similar NLS mutants for other flavivirus NS5 will also strengthen the evidence supporting a role for nuclear NS5 in general.

### IFN Signaling

Although flaviviruses actively exploit various strategies to suppress the production of IFN by infected cells, secreted IFN can still bind to the heterodimeric IFN receptor, IFNR1 and IFNR2 that are present on most cells. Binding of IFN to the receptors triggers the activation of JAK1 and Tyk2 to phosphorylate cytoplasmic STAT1 and STAT2 (Darnell et al., 1994). The phosphorylated STAT1 and STAT2 form a heterotrimeric complex with interferon regulatory factor 9 (IFR9) known as IFN-stimulated gene factor 3 (ISGF3), which translocate to the nucleus and binds to interferon-stimulated response element (ISRE) to regulate the transcription of IFN-stimulated genes (ISGs), many of which are antiviral (Haller et al., 2006).

Multiple studies have shown that flaviviruses use different ways to manipulate IFN signaling. DENV NS2A, NS4A, and NS4B block the IFN-induced transduction cascade in human A549 cells by interfering STAT1 phosphorylation, resulting decreased IFN-induced ISRE-promoter activation and enhanced DENV2 virus replication (Muñoz-Jordán et al., 2003, 2005). NS5 also inhibits IFN signaling *via* multiple mechanisms that appear to be virus-specific (Best et al., 2005; Guo et al., 2005; Lin et al., 2006; Park et al., 2007; Laurent-Rolle et al., 2010; Grant et al., 2016), and we will highlight mechanisms for which the role of NS5-host protein interactions has been dissected. NS5 of Langat virus (LGTV), a member of tick-born encephalitis complex of viruses, also interacts with IFN-a/b receptor subunit (IFNAR2) and IFN-g receptor subunit (IFNGR1) to block Jak1 and Tyk2 phosphorylation (Best et al., 2005; Park et al., 2007). TBEV NS5 protein also interacts with hScrib, a protein is expressed at the membrane of mammalian cells and controls cell-to-cell contact, resulting in impaired pSTAT1 formation in response to IFN-a/b and IFN-g (Werme et al., 2008). TBEV and WNV NS5 can also inhibit IFNAR1 mutation and accumulation at the cell surface through an interaction with PEPD (Lubick et al., 2015). DENV and ZIKV NS5 interact with and target STAT2 for proteasome-mediated degradation to inhibit IFN-induced signaling (Ashour et al., 2009; Mazzon et al., 2009; Morrison et al., 2013; Grant et al., 2016). Interestingly, DENV and ZIKV NS5 can target and degrade human STAT2 (hSTAT2) but not mouse STAT2 (Ashour et al., 2010; Grant et al., 2016). Thus, STAT2 is a species-specific target of a flaviviral nonstructural protein, similar to STING. Given the many emerging flaviviruses that circulate in non-human reservoirs, exploring the biophysical and biochemical differences underlying species-specific virus restriction could help predict the constraints to emergence.

### Complement System

The complement system is an important part of innate immunity to control early infection. It contains more than 50 plasma proteins and membrane proteins expressed on cell surface (Merle et al., 2015a). Complement activities occur in plasma, in tissues, or within cells (Kolev et al., 2014). The activation of complement happens through three distinct target-dependent pathways: classical, lectin, and alternative pathways. The classical pathway is initiated by the direct binding of C1q to the pathogen surface or antigen–antibody complexes; the lectin pathway is activated when mannose binding lectin (MBL), a serum protein, binds to mannose-containing carbohydrates on pathogens; and the alternative pathway is active when a spontaneously activated complement component binds to the pathogen surface (Merle et al., 2015a). Each pathway has its own protease to target and process different antigens, but they all generate a protease called C3 convertase and share common terminal outcomes after C3 cleavage: pathogen opsonization, regulation of inflammation, and clearance of immune complexes and cell debris. Complement activation is a bridge linking innate immune response and adaptive response by B cells and T cells in viral infection (Mehlhop and Diamond, 2006; Merle et al., 2015b).

Flaviviruses have evolved mechanisms to antagonize this part of the innate immune system. Among NS proteins from flaviviruses, NS1 has been recognized as an immune invasion protein that interferes with the complement system. A study by Avirutnan showed that NS1 from DENV, WNV, and YFV reduced complement activation pathways by interacting and forming a complex with C4 and C1s, leading to reduced classical pathway C4b deposition and C3 convertase (C4b2a) activity and consequent protection of DENV from complement-regulated neutralization (Avirutnan et al., 2010). In another study, NS1 from these flaviviruses directly binds to C4b binding protein (C4BP), a regulatory plasma protein of the classical and lectin pathway, to inactivate C4b in both cell surface and fluid; thereby protecting the viruses from complement attack (Avirutnan et al., 2011). WNV NS1 also binds to factor H (fH), a key regulator of the alternative pathway, and facilitates factor I-mediated cleavage of C3b. Additionally, cell surface-associated NS1 recruits fH and reduces C3b deposition and C5b–9 membrane attack complexes on cell surfaces, reducing the recognition of infected cells by complement system (Chung et al., 2006). For the lectin pathway, insect-derived DENV NS1 not only binds to human C1s, C4, and C4b-binding protein to suppress classical pathway of complement activation but also binds to mannose binding lection (MBL) to disrupt neutralization by the lectin pathway (Thiemmeca et al., 2016). DENV NS1 was also reported to interact with other proteins and interfere with the terminal pathway of complement activation. Specifically, NS1 interacts with complement regulator vitronectin (VN) and inhibits membrane attack complex (MAC) formation, suggesting a role of NS1 in antagonizing complement activation (Conde et al., 2016).

## Flavivirus-Host PPIs Involved in Disease

While identifying virus-host PPIs is important to inform on the fundamental mechanisms driving their replication, they can also be critical to understanding pathogenesis. Indeed, in recent years, several individual interactions have sparked interest in how flaviviruses alter host cellular behavior to cause disease. ZIKV has deservedly received significant research attention due to its unique association CZS. The most notable presentation of CZS is microcephaly, a condition in which head and brain size are dramatically reduced at birth (Moore et al., 2017). Many studies have provided insight into the mechanisms by which ZIKV causes CZS. Notably, however, is that many of these studies focus on virus strain/variants, placental damage, and the innate immune response *in utero* (Miner et al., 2016; Jagger et al., 2017; Rosenfeld et al., 2017; Yuan et al., 2017; Platt et al., 2018; Yockey et al., 2018). Here, we review how flavivirus-host PPIs directly dysregulate important developmental pathways to cause CZS.

In our own global proteomics screen, we identified an interaction between ZIKV NS4A and host ANKLE2 (Shah et al., 2018), mutations in which are known to be associated with hereditary microcephaly in humans and small-brain phenotypes in *Drosophila melanogaster* (flies; Yamamoto et al., 2014). NS4A expression alone in flies is sufficient to induce similar brain size defects in an ANKLE2-dependent manner. Further investigation revealed ANKLE2 is critical for spindle pole alignment during asymmetric division of fly neuroblasts, akin to mammalian neuroprogenitor cells that are targeted by ZIKV, and expression of NS4A results in similar division defects. Elegant fly genetics were used to demonstrate that NS4A inhibits the ANKLE2 pathway specifically (Link et al., 2019). Together, this demonstrates NS4A interacts with and disrupts ANKLE2 function, which in susceptible neuroblasts can disturb brain development. The extent to which this specific interaction impacts vertebrate brain development requires further investigation. Additionally, these studies bring to light the interplay between host genetics and viral pathogenesis. In flies, Ankle2 mutation heterozygosity results in normal brain development. However, NS4A expression in these flies is dramatically more severe than in wild-type flies (Shah et al., 2018; Link et al., 2019), suggesting that host genetics can pre-dispose an organism to disease that may be associated with virus-host PPIs. Another example of this phenomenon involves the previously discussed host-factor TMEM41B. Naturally occurring single nucleotide polymorphisms that lead to Ile266Val/Leu substitutions are prevalent in certain human populations but fail to rescue flavivirus replication in TMEM41B KO cells. This suggests these variants cannot be utilized by flaviviruses for the function they require to effectively replicate (Hoffmann et al., 2021).

One of the most common clinical findings associated with CZS is intracranial calcifications (Pool et al., 2019). A recent study explored how ZIKV induces these calcifications through the specific interaction between the viral protease NS3 and host bone morphogenic protein 2 (BMP2). BMP2 is an essential signaling protein in the process of osteogenesis, inducing the expression of downstream genes that ultimately facilitate bone growth. BMP2 normally must be cleaved by furin-type proteases prior to secretion, where it then induces these signaling cascades. Infection with ZIKV leads to increased expression of BMP2 and downstream genes, and subsequent calcification *in vitro* and *in vivo*. In fact, the expression of NS3 alone is sufficient to induce these phenotypes in U2OS osteosarcoma epithelial cells, but not a protease-defective mutant, suggesting that ZIKV NS3 cleavage of BMP2 initiates osteogenesis in the brain, leading to intracranial calcifications (Chen et al., 2021).

Beyond these examples, several other ZIKV-host interactions have been found to impact brain development and may ultimately play a role in human pathogenesis. Even while the ZIKV epidemic was ongoing it was shown that expression of ZIKV NS4A and NS4B specifically impaired the growth of NSCs by perturbing autophagy, while corresponding DENV proteins did not (Liang et al., 2016). This is not the only example of ZIKV-specific effects. Expression of ZIKV NS2A *in vivo* disrupts neurogenesis through physical interactions with adherens junctions in radial glial cells (Yoon et al., 2017). In another recent study, systematic proteomics in NSCs revealed the interaction between ZIKV Capsid and Dicer, a pivotal protein in the host RNAi pathway with implications in neurodevelopment (Zeng et al., 2020). Among flaviviruses, this interaction is also unique to ZIKV. Mechanistically, Dicer is a host restriction factor and ZIKV Capsid interaction inhibits this antiviral function, as infection with a H41R mutation, which ablates this interaction leads to less viral burden *in vivo*. Indeed, even ZIKV Capsid expression alone, dependent on its interaction with Dicer, is sufficient to induce severe defects in brain development.

Together, these studies highlight how certain aspects of pathogenesis may be uniquely derived from single virus-host PPIs. However, given the incredible complexity of human development, it is not likely that any single interaction during infection is solely responsible for disease outcome. More realistically in the case of ZIKV, it is the culmination of these perturbations and dysregulation of brain development by multiple mechanisms that results in CZS. Intriguingly, the intersection of flavivirus-host PPIs and disease, including host factors implicated in hereditary disease, opens the door to the possibility of host genetics being a major and overlooked contributing factor to susceptibility to CZS. For example, loss-of-function variants for host factors like ANKLE2, which are haplo-sufficient for their role in development in the absence of a virus-host PPI, but haplo-insufficient in the context of a virus-host PPI, could tip the balance in the favor of disease in an otherwise healthy individual. Future studies exploring this concept are warranted.

## Concluding Remarks

Flaviviruses are arthropod-borne viruses that cause significant human disease worldwide. Their limited genome requires them to co-opt host proteins through physical interactions during infection to properly replicate. Some of these interactions appear to be broadly conserved among flaviviruses, while other unique interactions contribute to observed differences in host tropism and pathogenesis. Flaviviruses employ a wide range of host receptors utilized for entry into host cells. Replication within the ER involves vast remodeling into a microenvironment well-suited to the generation of viral progeny. Even outside the ER viral proteins orchestrate modulation of host cell systems. This includes physical interaction with other cellular pathways and organelles critical to virus replication and with different protein components of the host innate immune system. These virus-host PPIs can be influential in the development of pathogenesis. Thus, understanding these mechanisms is essential for creating new therapeutics to alleviate human disease caused by flaviviruses.

## Author Contributions

AF, OP, NB, MK, and PS wrote manuscript. MK, AF, OP, and NB designed and generated the figures. PS secured funding for work. All authors contributed to the article and approved the submitted version.

## Funding

Funding was provided by the W. M. Keck Foundation to PS and a NIH T32 Fellowship (2T32AI060555-16) to AF.

## Conflict of Interest

The authors declare that the research was conducted in the absence of any commercial or financial relationships that could be construed as a potential conflict of interest.

## Publisher’s Note

All claims expressed in this article are solely those of the authors and do not necessarily represent those of their affiliated organizations, or those of the publisher, the editors and the reviewers. Any product that may be evaluated in this article, or claim that may be made by its manufacturer, is not guaranteed or endorsed by the publisher.
